# Heat stress induced piRNA alterations in pachytene spermatocytes and round spermatids

**DOI:** 10.1186/s12958-024-01249-z

**Published:** 2024-07-24

**Authors:** Poonam Mehta, Shruti Sethi, Santosh Kumar Yadav, Gopal Gupta, Rajender Singh

**Affiliations:** 1 Division of Endocrinology, CSIR-Central Drug Research Institute, Lucknow, India; 2 Academy of Scientific and Innovative Research (AcSIR), Ghaziabad, India

**Keywords:** piRNA, Heat stress, Pachytene spermatocytes, Round spermatids, Male infertility

## Abstract

**Background:**

Spermatogenesis is a temperature-sensitive process, and elevation in temperature hampers this process quickly and significantly. We studied the molecular effects of testicular heating on piRNAs and gene expression in rat testicular germ cells.

**Methods:**

We generated a cryptorchid rat model by displacing the testis from the scrotal sac (34 °C) to the abdominal area (37 °C) and sacrificed animals after 1 day, 3 days, and 5 days. Pachytene spermatocytes and round spermatids were purified using elutriation centrifugation and percoll gradient methods. We performed transcriptome sequencing in pachytene spermatocytes and round spermatids to identify differentially expressed piRNAs and their probable targets, i.e., TE transcripts and mRNAs.

**Results:**

As a result of heat stress, we observed significant upregulation of piRNAs and TE transcripts in testicular germ cells. In addition to this, piRNA biogenesis machinery and heat shock proteins (Hsp70 and Hsp90 family members) were upregulated. mRNAs have also been proposed as targets for piRNAs; therefore, we shortlisted certain piRNA-mRNA pairs with an inverse relationship of expression. We observed that in testicular heat stress, the heat shock proteins go hand-in-hand with the upregulation of piRNA biogenesis machinery. The dysregulation of piRNAs in heat-stressed germ cells, increased ping-pong activity, and disturbed expression of piRNA target transcripts suggest a connection between piRNAs, mRNAs, and TE transcripts.

**Conclusions:**

In heat stress, piRNAs, piRNA machinery, and heat shock proteins are activated to deal with low levels of stress, which is followed by a rescue approach in prolonged stressaccompained by high TE activity to allow genetic mutations, perhaps for survival and adaptability.

**Supplementary Information:**

The online version contains supplementary material available at 10.1186/s12958-024-01249-z.

## Background

In 1950, Barbara Mclintock identified ‘mutable loci’ or ‘jumping genes’, which were linked with phenotypic variations in maize [1]. Initially, these elements were named as junk or selfish elements, which replicate using the host’s machinery. Further research revealed their biological significance in genome evolution, the maintenance of genetic diversity, and the regulation of gene expression. These elements are now called transposons or transposable elements (TE), and they have the potential to lead to deleterious mutations or advantageous genetic diversity. Therefore, a balance between transposable element activity and their repression has to be maintained. Accordingly, the genomes of various organisms have evolved machineries to regulate TE activity. PIWI (P-element-induced wimpy testis)-interacting RNAs (piRNAs) belong to the animal-specific class of small RNAs with a size range of 24–32 nt and a 2’-OMe modification on the 3’ end. PiRNAs are generated from either the TE transcripts or their specific loci (constituted by dysfunctional remnants of TEs) [2]. The mapping studies of piRNAs onto the genome have identified dense mapping of piRNAs to certain regions, which are defined as the piRNA clusters [3, 4]. PiRNAs originate primarily from the intergenic regions, transposable elements, pseudogenes, and protein-coding genes [5, 6] and have roles in development, transposon silencing, and the maintenance of genomic integrity [7].

Retro transposition occurs actively during cell division [8]. Due to massive cell divisions in the testis, the burden of retro transposition is very heavy on germ cells. Interestingly, piRNAs were first discovered in Drosophila male germ line [9]. During the active phase of cell division, the regulatory control for retro-transposition is piRNA-mediated recognition of the transposable elements and their repression by chromatin modifications [10]. Both piRNAs and piwi proteins (required for piRNA activity) are abundantly expressed in germ cells, suggesting their significant participation in spermatogenesis [11]. Among several possible functions of piRNAs in germ cells, the regulation of TE activity appears to be at the forefront to ensure genomic stability and faithful transmission of genetic information to the next generation. Heat shock in general represses global transcription [12], but the transcription of piRNAs and heat shock proteins is upregulated, suggesting their role in heat shock management in the germ cells. In Drosophila, it has been shown that heat shock modulated the expression of several piRNA clusters [13] and resulted in a burst of transposition [14]. The underlying mechanism in Drosophila involves the action of Hsp90 chaperone, which guides the AGO3 bound protein complexes to lysosomal degradation, thus increasing the expression of TEs by affecting piRNA biogenesis [15]. The activation of the stress management machinery in the testis even in higher organisms is well known [16], which helps in the transient management of stress until the return of favourable conditions.

It is well known that spermatogenesis takes place at 3–4 °C less than the body temperature. However, the molecular reasons behind this interesting phenomenon remain unknown. In mice, scrotal exposure to 40 °C or above temperatures results in hypoxia and oxidative stress-induced DNA damage response, an increase in germ cell death, and subfertility [17]. Interestingly, exposure to body temperature results in rapid arrest of testicular cells division, leading to azoospermia [18]. We undertook the present study to investigate the molecular players in response to testicular heat stress. To simulate the natural heat stress condition seen in cryptorchidism, we generated a cryptorchid rat model of heat stress. We hypothesized that a stress stimulus such as heat shock may affect piRNA machinery to alter gene expression as a rescue mechanism. Additionally, we also predicted the mRNA targets of piRNAs, followed by in vitro functional analysis.

## Materials and methods

### Animals

Adult male Sprague Dawley (SD) rats, aged 3–4 months and within a weight range of 200–250 g, were used in this study. The animals were kept in an air-conditioned GLP certified animal house facility with a temperature of 24 ± 1 °C, humidity of 60%, and a 12 h light and 12 h dark cycle with an ad libitum supply of food and water. The study was approved by the Institutional Animal Ethics Committee of the CSIR-CDRI [IAEC/2014/49/Renew03(135/16) and IAEC/2021/94/Renwer-0].

### Cryptorchidism/heat stress model generation

To generate the heat stress model, rats were anesthetized using a 3:1 dose of ketamine and xylazine. We placed the anesthetized rat in the ventral position, cleaned the hair from the abdominal area, and wiped the area with 70% ethanol. We made a cut in the abdominal area with a fine sterile scissor and pushed both the testicles from the scrotal sac to the abdomen. The testicles were held with the help of forceps and tied to the abdominal wall with the help of sutures. Sham operated animals served as controls and each group consisted of five animals. The model animals were sacrificed after one (named 1DCR), three (named 3DCR) and five (named 5DCR) days.

### Isolation and purification of pachytene spermatocytes and round spermatids

We used the centrifugal elutriation protocol, followed by percoll density gradient centrifugation for the purification of pachytene spermatocytes and round spermatids from rat testes [19]. We pooled all the five testes available from one group for the isolation of germ cells. Testes were decapsulated in such a way that the testicular artery was removed intact and the tissue was properly minced in 100 ml of Basal Medium Eagle (BME) supplemented with 0.1% trypsin, 0.1% glucose and 17 μg/ml DNase I. The minced testes tissue was incubated at 34 °C with continuous shaking in water bath for 15 min. The reaction was stopped by adding soyabean trypsin inhibitor (0.04% w/v). The cell suspension was filtered through a nylon mesh to remove debris and passed through glass wool to remove spermatozoa. The cell suspension was centrifuged at 400*g for 5 min at 4 °C and washed twice with the BME medium. Further, the cell pellet was resuspended in the BME medium supplemented with DNaseI (2 μg/ml) and fetal bovine serum (8% v/v).

For the separation of germ cells on the basis of their size, we used a high-speed centrifuge with a JE-5 rotor and a standard chamber where cells experience centrifugal and flow rate forces and get separated accordingly (Beckman Coulter, California, US). We first cleaned the inlet, outlet and chamber by passing 70% ethanol, followed by rinsing with water and a final rinse with the BME medium. We maintained the centrifuge at 4 °C and injected the cell suspension using a syringe in the inlet glass tube. We collected four fractions and labelled the tubes as Ia and Ib for fraction I, IIa and IIb for fraction II, IIIa and IIIb for fraction III, and IVa and IVb for fraction IV. The centrifugal force and flow rate settings for each fraction are mentioned in Table 1.

**Table 1:** Elutriator centrifugal and flow rate settings

Fraction	Centrifugal force	Flow rate	Pump reading
I	3000 RPM	18 ml/min	0.70
II	3000 RPM	31.5 ml/min	1.04
III	2000 RPM	23 ml/min	0.80
IV	2000 RPM	40 ml/min	1.20

For each fraction mentioned above, 50 ml volume was collected in each tube according to the above conditions. We centrifuged the collected fraction tubes at 400*g for 5 min at 4 °C and observed the cells under a microscope. Fraction IIb contains purified round spermatids and fraction IVb contains purified pachytene spermatocytes. These fractions contained spermatocytes and spermatids pure up to 80%. The next step of purification was performed with percoll gradient centrifugation. We prepared two gradient vials, one with 22–32% for round spermatids and another with 25–35% for pachytene spermatocytes, and centrifuged at 4025*g for 60 min in a swinging bucket rotor. The cells were collected by puncturing the side of the tube, followed by washing with the BME medium. Percoll gradient purification provided us with ∼ 90% pure spermatocytes and spermatids.

### RNA isolation, quality check and polyA enriched RNA library preparation

Total RNA was isolated using the Qiagen RNeasy Micro kit (Cat. No. 74004). RNA integrity was checked using the RNA6000 pico kit (Cat. No. 5067 − 1513) on an Agilent 2100 Bioanalyzer. The samples with RIN values > 8 were considered good for library preparation. Poly A enrichment of RNA was done using the Dynabeads mRNA DIRECT™ kit (Cat. no. 61012, Life Technologies) and library preparation was undertaken using the TruSeq RNA kit (RS-122-2001, Illumina). We followed the procedure as detailed in the manual.

### Small RNA library preparation

For small RNA library preparation, the NEBNext Multiplex Small RNA library prep kit (Cat. No. E7560S) was used. 100 ng of the total intact RNA was used as the input material. The libraries were prepared as instructed in the manual, except for a few changes at some steps. We did not perform PCR purification using the Monarch kit, against what was suggested in the manual. The total volume after PCR was directly loaded on a 6% PAGE gel for size selection of the libraries. The small RNA libraries corresponding to 140–160 nucleotides were excised and purified from the gel. The purified libraries were checked on an Agilent 2100 bioanalyzer using a high-sensitivity DNA kit (5067 − 4626) for concentration and size confirmation.

### Sequencing and data processing

Single-end sequencing was undertaken on the Illumina Hiseq 2500 platform with an average read length of 75 nucleotides. For paired analysis of piRNAs and target gene expression, we also used transcriptome data generated in our previous study [20]. A FastQC check was performed on the raw data. The quality control passed samples were further processed for data analysis.

### TE transcript expression analysis

The TE transcripts tool was used for the identification of unit length TE transcripts from polyA enriched RNA sequencing [21]. The TE transcripts tool takes two input files, i.e., BAM file generated after alignment and GTF (General transfer format) file of genes and TE transcripts. RNA seq fastq files were processed using the FASTX tool, followed by alignment with the rat reference genome rn6 using the bowtie2 aligner. The SAM file thus generated was converted to BAM using samtools. Another input file, i.e., GTF file for TE, was generated from Repeatmasker (http://www.repeatmasker.org). Since TE transcripts can have multiple origins due to their repetitive nature, a multi-read approach was used in the case of TE transcripts. Differential expression analysis of the TE transcripts was undertaken using the DESeq2 R package.

### Transcriptome data analysis

The processed reads were aligned to the genome using an RNA sequencing-specific aligner, i.e., Tophat (https://ccb.jhu.edu/software/tophat/index.shtml). For further analysis, we used the cufflinks suite (http://cole-trapnell-lab.github.io/cufflinks/). The output bam file from the aligner was used as input for cufflinks to assemble the reads into transcripts, and cuffdiff (http://cole-trapnell-lab.github.io/cufflinks/) was used for differential expression analysis of transcripts.

### piRNA analysis and the origin of piRNAs

The FASTX tool (http://hannonlab.cshl.edu/fastx_toolkit/) was used for data processing, which includes removing the 3’adapter sequences and read length filtering. For piRNA analysis, 24–35 nucleotide read length unique sequences were filtered and converted into the FASTA format. These sequences were subjected to a homology search against the rat piRBase database (http://bigdata.ibp.ac.cn/piRBase/) and perfectly mapped sequences with a minimum of 10 reads were considered confident piRNAs. To define repeat-derived piRNAs, the Rn6 repeat masker output file was used for mapping using the blast tool. In cases of multiple hits, the best hit was chosen. This allowed us to identify repeat-associated piRNAs while others were considered non-repeat derived piRNAs. The differential expression analysis of piRNAs was done using the DESeq2 median of ratios method of normalization (https://bioconductor.org/packages/release/bioc/html/DESeq2.html).

### piRNA cluster prediction and characterization

proTRAC (version 2.4.3) was used for cluster prediction [22]. This applies a sliding window approach to detect loci that exhibit high sequence read coverage. The minimum size of clusters was considered to be 1000 bp. The clusters were predicted using the default settings of a sliding window size of 5000 bp, normalizing the hits by the number of sequencing reads. The genome assembly used for extracting the location was Rn6 from the UCSC genome browser.

### Ping-pong amplification analysis

The PPmeter tool was used to quantify and compare the ping-pong amplification [23]. It generates pseudo-replicates by bootstrapping the fixed number of reads from the original set of sRNA sequences. It calculates the ping pong signature for each pseudo-replicate and counts the reads that participate in ping-pong amplification. The final outcome is ping-pong reads per million bootstrapped reads, which is directly comparable across different datasets.

### piRNA target prediction

piRNA target prediction was undertaken considering the rule of minimum 16 nucleotide complementarity to the target [24]. The targets were predicted for the 3’UTR, 5’UTR, and CDS of the mRNA transcripts. The Miranda tool was used to define the targets of piRNAs with a cut-off score of 150, and binding energy of at least − 20 kcal/mol.

### In vitro piRNA transfection and target validation

For functional in vitro assays of piRNA targets, we selected one upregulated (piR-rno-41911) and one downregulated piRNA (piR-rno-588062). Chemically synthesized piRNA sequences with 2’O methyl modification at the 3’ termini and the cy3 label at the 5’end were purchased from Integrated DNA Technologies (IDT).

We conducted in vitro assays using two approaches. In the first approach, the piRNAs were transfected in the GC2 cell line (CRL-2196) at 100 nM concentration using the Xfect RNA transfection reagent (Cat. No. 631450, Takara) and the scrambled sequence was used as a negative control.

In another set of in-vitro experiments, we generated stably transfected cell lines using the pSCALPS-puro-EGFP-MIWI vector (kindly gifted by Dr. Phillip D. Zamore), facilitating the delivery of the MIWI transgene into GC-2 cells. Specifically, the transfer plasmid was paired with a second-generation envelope plasmid (pMD2.G) and a second-generation packaging plasmid (psPAX2). The transfer plasmid in the lentiviral vector system of the third generation incorporated a viral backbone containing puromycin-resistant gene and a GFP coding sequence, strategically designed to streamline the selection process for cell lines. GC-2 cells were then infected with MIWI-containing lentivirus using protamine sulfate. Following transduction, media supplemented with 1 μg/ml puromycin was administered after 24 h to facilitate cell selection. Subsequently, GFP-tagged cells were isolated using the BD FACSAria™ III Cell Sorter to confirm successful transduction of lentiviral particles in GC-2 cells. These GFP-tagged cells were subsequently cultured through 5–6 passages in puromycin-containing media to ensure stable integration of the transgene into GC-2 cells. Cells were also observed under a fluorescence microscope to check GFP expression and further confirm the transduction of cells with lentivirus particles. Further, to check the efficiency of MIWI transgene incorporation in cell lines, the expression of MIWI in the transduced cells was measured with qRT-PCR. MIWI expressing GC-2 cells were transfected with piR-588062, piR-41911, and scrambled sequence at a concentration of 100 nM/ml using Xfect™ RNA transfection reagent (Takara Bio USA, Inc. United States/Canada). MIWI stable GC-2 and non-transduced GC-2 cells were cultured in 6-well plates until reaching 70% confluency. A scrambled sequence was utilized as a control. Following four hours of transfection, Opti-MEM was replaced with complete medium, and cell culture was terminated after 48 h.

In both of the above sets of experiments, 48 h after transfection, we washed the 6-well plate with DPBS, observed the cells under a microscope for cy3 positivity, and proceeded with cell scraping and RNA isolation using Trizol. We checked the piRNA and target gene expression levels before and after transfection. The levels of the target transcripts were quantified using the RT-qPCR method and compared with a negative control. The complete list of chemically synthesized sequences is given in Supplementary Table 1.

### qRT-PCR analysis

For qRT-PCR analysis, RNA was isolated from the transfected cells using the Trizol method. Subsequently, 1000 ng of RNA was reverse-transcribed into cDNA using Applied Biosystem’s high-capacity cDNA conversion kit (Cat. no. 4368814). The resulting cDNA was subjected to qRT-PCR to assess transfection efficiency by analysing the levels of piRNAs in transfected cells. The relative expression of the housekeeping gene U6 was utilized for normalization. Primers designed for target mRNA expression analysis were used with Platinum SYBR Green from Applied Biosystems, following a pre-determined protocol. Additionally, the relative expression of the housekeeping gene b-actin was employed to normalize the expression of the target gene.

## Results

### Cryptorchid model and isolation of pachytene spermatocytes and round spermatids

**Fig. 1:**
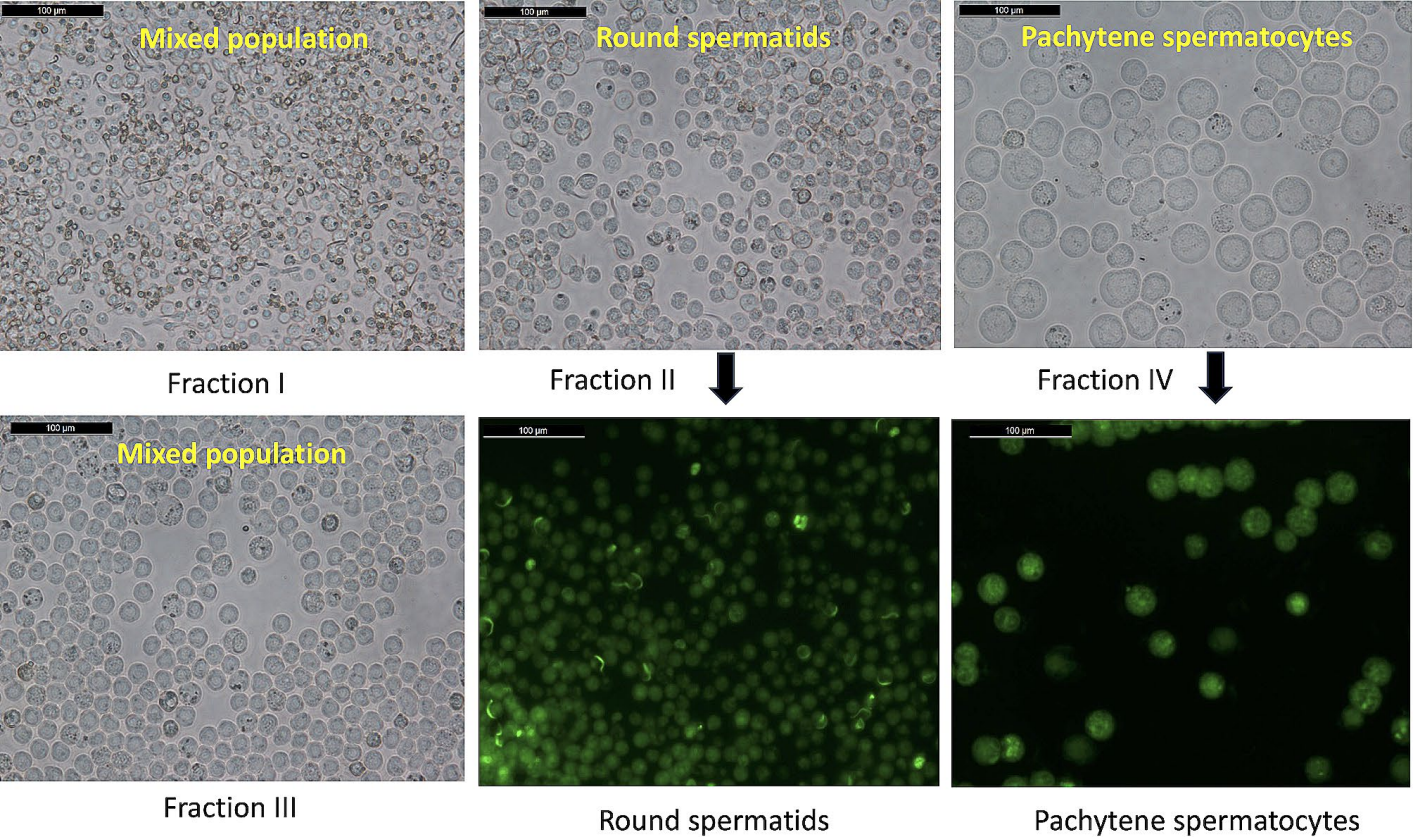
Germ cell preparation: Cell fractions collected after centrifugal elutriation and acridine orange stained purified pachytene spermatocytes and round spermatids after centrifugal elutriation and percoll gradient

As shown in our previous study, H&E staining showed no visible change after one day of heat stress, but significant changes were observed after three and five days of heat stress [20]. The testicular cell population was significantly reduced by the 3rd and 5th days, the testicular architecture was distorted, and the germ cells were seen dislodging and flowing out in the lumen area. Thus, an increase of three degrees of temperature can inflict significant damage upon the testis, and this can be used as a model for studying spermatogenesis. The collected testis samples from normal and heat-stressed rats were processed for the isolation of germ cells using centrifugal elutriation. Four collected fractions were observed under a bright field microscope to check for cell populations enriched in each fraction (Fig. 1). Round spermatids were found in the second fraction and pachytene spermatocytes were found in the fourth fraction. The desired fractions were further taken for Percoll gradient centrifugation to increase the purity of the cells. The purified cells were stained with acridine orange and observed under a fluorescent microscope (Fig. 1).

### Sequencing data pre-processing and quality analysis

We generated 25–30 million reads for small RNA sequencing, and 96% of the bases had phred scores equal to or greater than 30. The alignment statistics for the samples represented 90% or above alignment. In the case of small RNAs, we were left with 5–20 million reads after adapter trimming, data processing, and read length filtering (24–35 nt). The percent distribution of the reads, along with length information, is presented in Supplementary Fig. 1. The reads corresponding to 29 and 30 nucleotides constituted the major fraction in both spermatocytes and spermatids. However, another major peak seen at 26 nucleotides in the case of spermatocytes disappeared in spermatids. A previous study on piRNA sequence length showed that the major peak in spermatocytes was at 26 nucleotides, which changed to 30 in the case of spermatids [25].

### Characterization and chromosomal distribution of piRNAs in pachytene spermatocytes (PS) and round spermatids (RS)

**Fig. 2:**
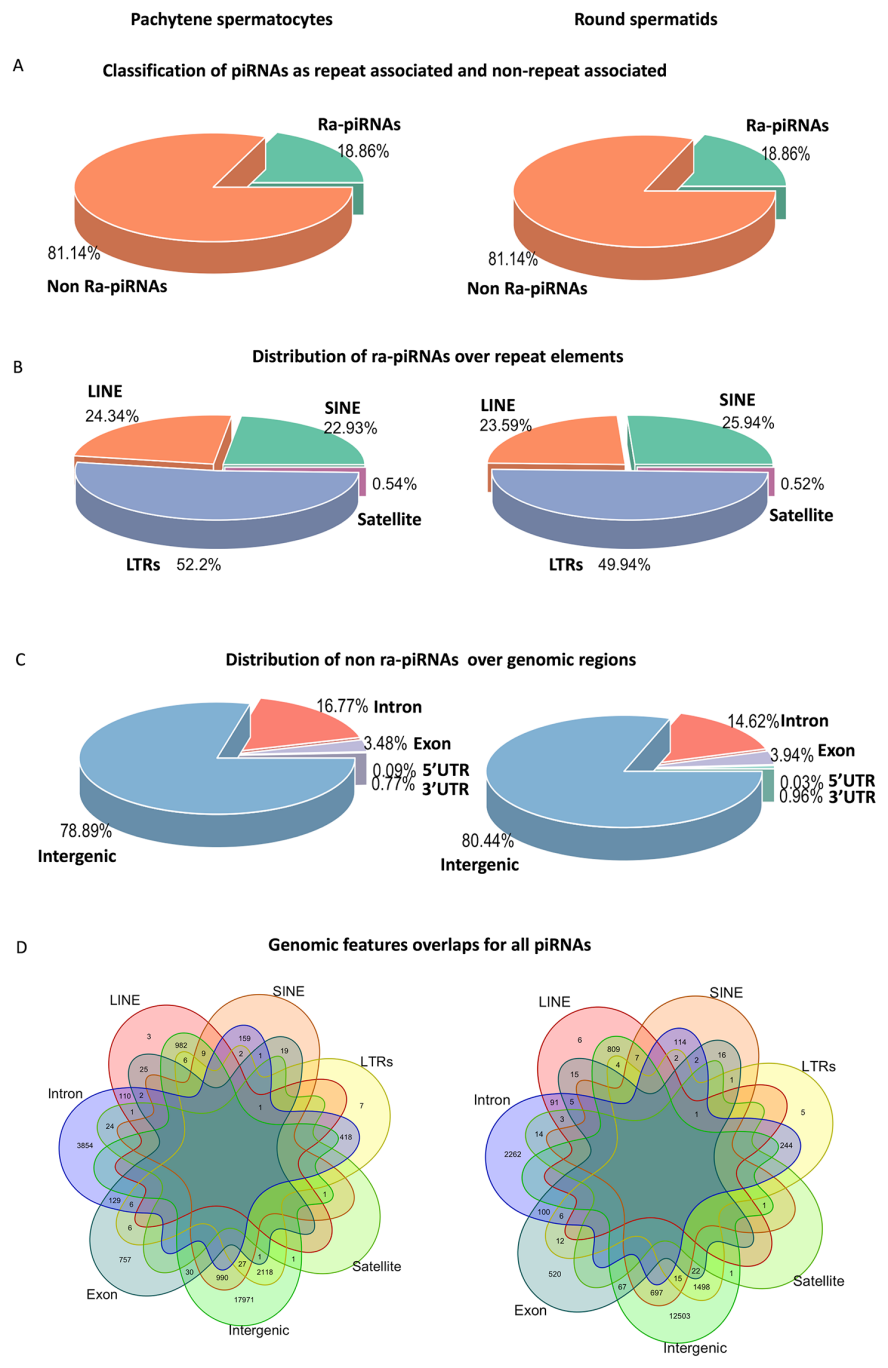
piRNA classification: Classification of piRNAs into repeat-associated and non-repeat associated (**A**), the distribution of repeat-associated piRNAs into various repeat elements (**B**), the distribution of non ra-piRNAs into various genic features (**C**), the classification of all piRNAs into various genomic features (**D**)

Based on 100% homology to database sequences, we identified confident piRNAs after applying a minimum read count filter of 10. Due to their repeat associated nature, we mapped piRNAs to repeat sequences to determine the origin of the pachytene spermatocyte and round spermatid piRNA sequences. We classified the sequences as repeat-associated and non-repeat-associated piRNAs. In both pachytene spermatocytes and round spermatids, the majority of piRNAs (∼ 82% and 81%, respectively) were non-repeat derived and only a small fraction was repeat derived (18% and 19%, respectively) (Fig. 2A). The majority of the repeat derived piRNAs in both the cell types were LTR-derived, followed by LINE and SINE derived, and a minor fraction was derived from satellite regions (Fig. 2B). Non-repeat derived sequences were further screened for their origin from various genic regions, such as 5’UTR, exon, intron, 3’UTR, and intergenic regions. We found that the majority of the sequences (∼ 79% in PS and ∼ 80.44% in RS) were intergenic region-derived (Fig. 2C). This is in accordance with previous observations of a high abundance of intergenic derived piRNAs in spermatocytes and spermatids [25]. Due to the overlapping nature of genomic annotations, we also plotted their overlaps and identified that the majority of sequences mapped exclusively to the intergenic regions of the genome (Fig. 2D), suggesting that piRNAs in pachytene spermatocytes and round spermatids are derived primarily from intergenic regions. To rule out false positive predictions for the distribution of piRNAs, we also repeated this for sequences with 100 plus reads. However, the percentage trend in the subgroups remained the same.

**Fig. 3:**
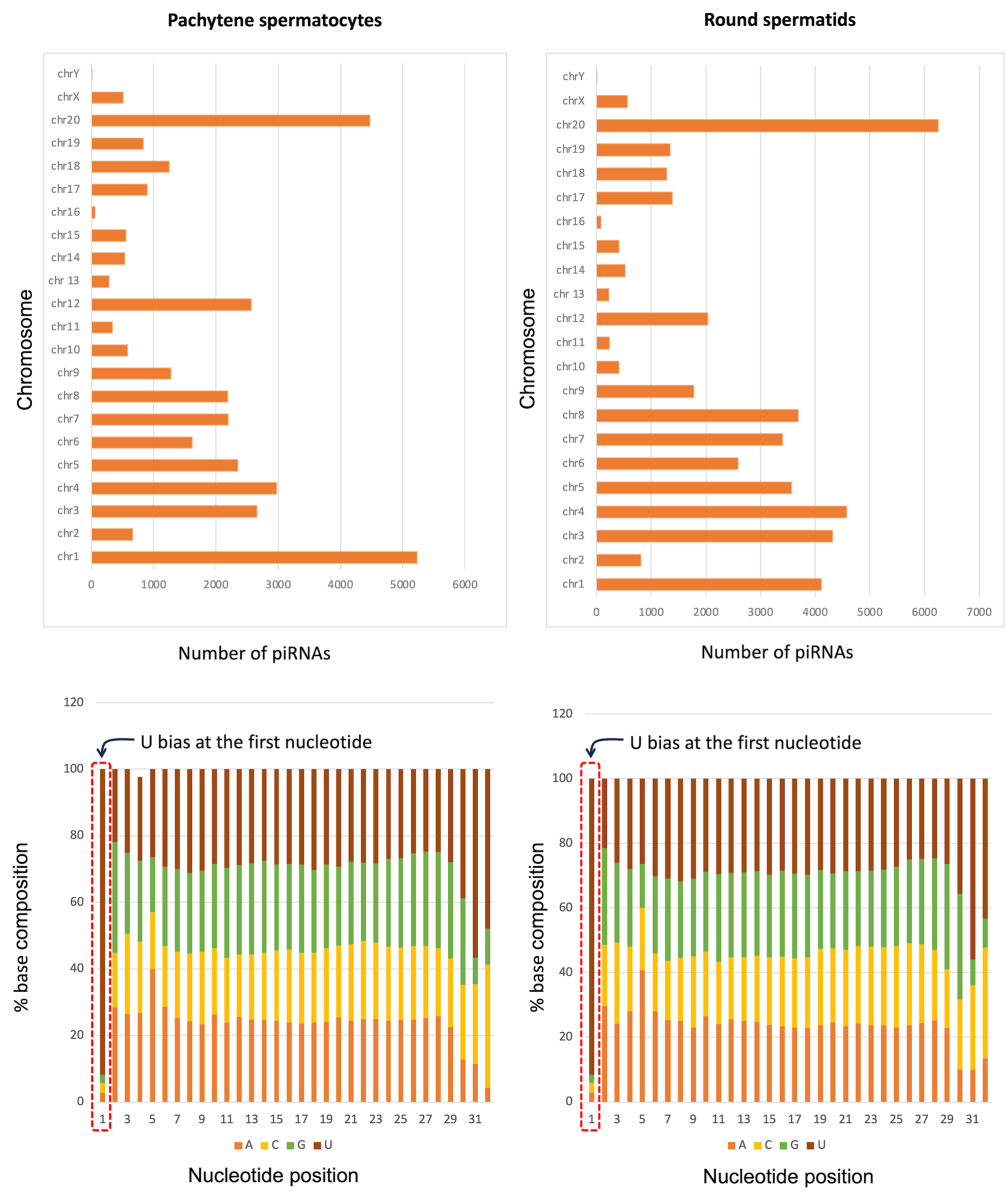
Characteristic features of piRNAs: Chromosomal distribution of all piRNAs in spermatocytes and spermatids (upper panel), percent nucleotide composition of all piRNA sequences as per the nucleotide position (lower panel)

The chromosomal distribution of the piRNAs showed that the maximum number of piRNAs originated from chromosomes 1, 3, 4, 12, and 20 (Fig. 3A). The nucleotide distribution for the sequences possessed 5’ 1U-bias, which is mostly harboured by piRNAs (Fig. 3B). We also checked the possibility of another annotation that can be conferred to the intergenic regions. Therefore, we mapped the piRNA data to the long non-coding RNAs and found that 16.96% of piRNAs that mapped to the intergenic regions, also mapped to lncRNAs. In the case of the lncRNAs mapping outcome, we found that 156 piRNAs with 100 or more abundances mapped to 34 lncRNAs. An average of 13–17 piRNAs with abundances of ≥100 mapped to five major lncRNAs transcripts, i.e., ENSRNOT00000094412.1, ENSRNOT00000094584.1, ENSRNOT00000109391.1, ENSRNOT00000110707.1, and ENSRNOT00000120048.1 (Supplementary Fig. 2). All these lncRNAs were regarded as novel transcripts in the Ensemble genome browser.

The piRNA sequences that did not map to the database with 100% homology were considered novel sequences. The novel sequences (*n* = 15,536 with a read depth of 100 or above) in the length range of 24–35 nt were also subjected to percent base composition screening to identify the sequences with piRNA 5’1U bias. Interestingly, we found 14,337 sequences (∼ 92%) with U at the first position. These additional sequences, just like piRNAs, have 5’U bias, and the probable reason for their failure to map to the database sequences was the less conserved nature of piRNAs. These sequences were subjected to mapping to identify their origins. Similar to confidently mapped piRNAs, these potential piRNAs were largely of intergenic origin. Their independent mapping on repeats and lncRNAs identified 16.06% and 3.25% mapping, respectively.

### piRNA cluster identification

**Fig. 4:**
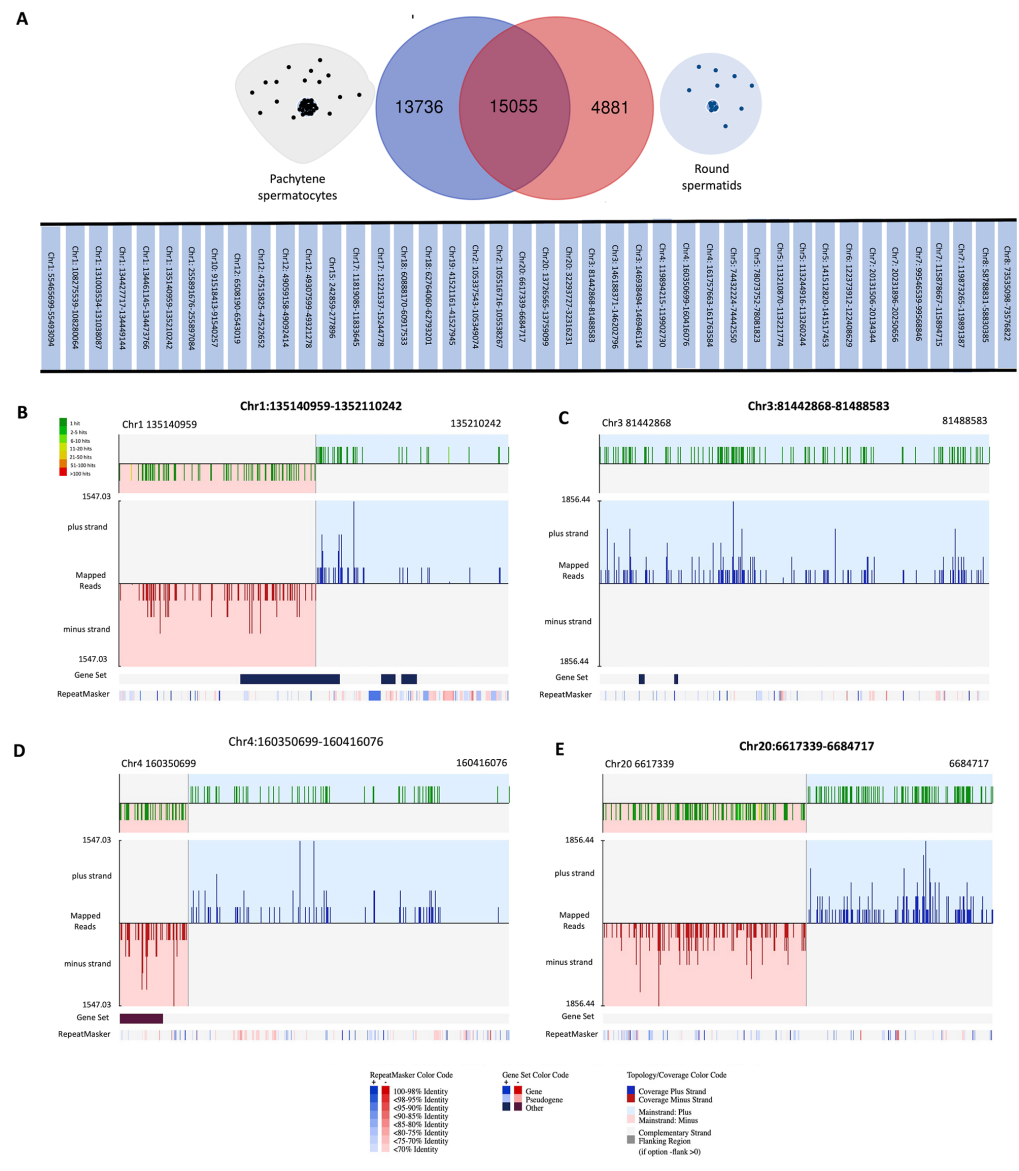
piRNA clusters predicted using proTRAC: piRNA clusters common in pachytene spermatocytes and round spermatids (**A**), Four biggest piRNA clusters in pachytene spermatocytes were present on chromosome 1 (**B**), chromosome 3 (**C**), chromosome 4 (**D**), and chromosome 20 (**E**)

Since piRNAs are transcribed as large precursor RNAs, followed by their cleavage into mature piRNAs, it is important to know the cluster organization of germ cell piRNAs. proTRAC (probabilistic Tracking and Analysis of Clusters) predicted 63 piRNA clusters in pachytene spermatocytes and 57 clusters in round spermatids. The comparison of the piRNA clusters in the two cell types revealed 42 common clusters (Fig. 4A) and 21 clusters unique to spermatocytes and 15 clusters unique to round spermatids. The complete list of predicted clusters along with their chromosomal coordinates is presented in Supplementary Table 2.

The four biggest clusters common to both cell types were; Chr1:135140959–1352110242, Chr3:81442868–81488583, Chr4:160350699–160416076, and Chr20:6617339–6684717 (Fig. 4B-E). The syntenic clusters to the above four in the mouse genome are located on Chr7 (Chr7:73773862–73816453; Chr7:73816505–73834434), Chr2 (Chr2:92541187–92592844), Chr6 (Chr6:127768903–127796380; Chr6:127796460–127823093), and Chr17 (Chr17:27310318–27324774; Chr17:27325406–27348670), respectively. Similarly, we performed cluster prediction for heat-stressed spermatocytes. We identified 20 unique clusters in heat-stressed spermatocytes (Supplementary Table 3). It suggests the expression of piRNAs from a higher number of clusters (perhaps reserve) under heat stress conditions.

### Differential piRNA expression profiles of spermatocytes and spermatid in heat stress model

**Fig. 5:**
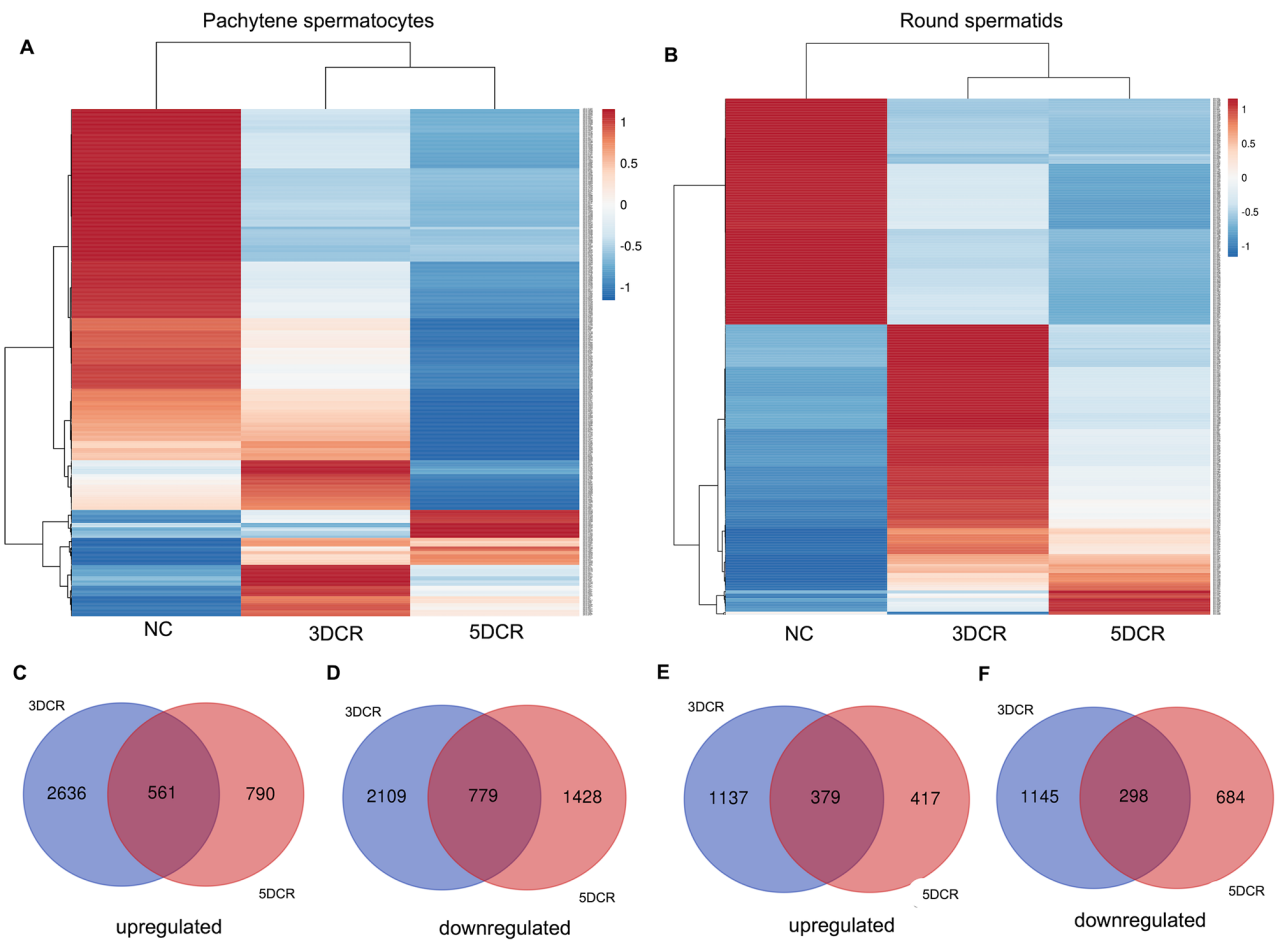
Differential expression of piRNAs in heat stress: heatmap visualization of differentially expressed piRNAs in spermatocytes (**A**) and spermatids (**B**). Venn diagram for commonly upregulated and down-regulated piRNAs (**C**-**F**)

The piRNAs with 10 plus abundances were taken forward for differential expression analysis. Heat map comparisons across NC, 3DCR and 5DCR spermatocytes and spermatids showed that gene expression profiles changed drastically from NC to 3DCR and 3DCR to 5DCR (Fig. 5A and B). The differential expression analysis in NC spermatocytes versus 3DCR spermatocytes showed 6.86% upregulated piRNAs, 6.18% downregulated piRNAs, 14% piRNAs unique to NC spermatocytes, 38.48% (*n* = 18,104) unique to 3DCR spermatocytes and 34% remained unaltered. About 18,000 sequences that appeared in heat-stressed spermatocytes only were analysed for their origin, which showed that a major chunk was intergenic-derived (61.5%), only 20.16% were repeat-derived and the rest were exon/intron/UTR region-derived. However, among the additional sequences that did not map to the database sequences, 42.3% were repeat-derived and 39.3% were intergenic-derived. Similarly for round spermatids, the differential piRNA analysis showed 5.05% upregulated, 4.81% downregulated, 15% unique to NC spermatids, 33.3% of sequences unique to 3DCR spermatids and 41.5% remain unaltered. The common dysregulated piRNAs from 3DCR and 5DCR pachytene spermatocytes (Fig. 5C and D) and round spermatids (Fig. 5E and F) were picked for downstream analysis of piRNA targets under heat stress.

### Ping-pong amplification of piRNAs under heat stress

**Fig. 6:**
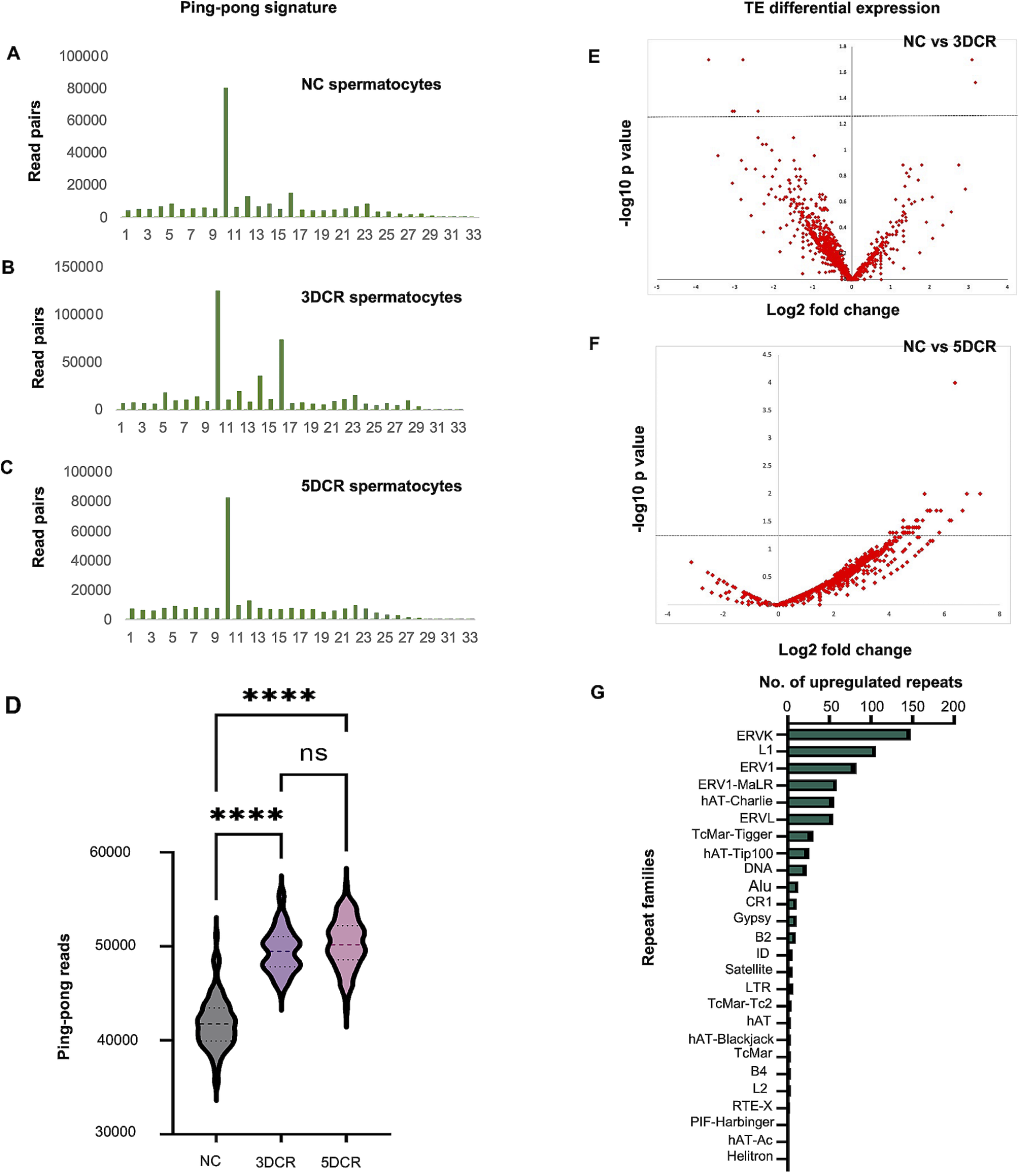
Ping-pong signature and TE expression analysis: Ping-pong signatures were identified in the small RNA read pairs in normal spermatocytes (**A**) 3DCR spermatocytes (**B**) and 5DCR spermatocytes (**C**) Normalized ping pong reads comparison in NC, 3DCR and 5DCR (**D**), TE transcripts differential expression in NC vs. 3DCR (**E**) NC vs. 5DCR (**F**), upregulated repeat families in 5DCR spermatocytes (**G**)

PPmeter identified the ping pong signatures and counted the read pairs participating in ping pong activity (Fig. 6A-C). This data is calculated from bootstrapped datasets; therefore, the data was comparable across different datasets. Heat-stressed spermatocytes showed increased ping-pong activity when compared to normal spermatocytes (Fig. 6D), but it was not observed in spermatids (data not presented). Therefore, in heat-stressed spermatocytes, there is an amplification of silencing, which may have increased in response to an increased activity/expression of transposons. Interestingly, piRNAs are also known to be generated by a non-canonical ping-pong pathway [25].

### Disturbed TE expression in heat stress

Next, we analysed the differential expression of TE transcripts in normal and heat stressed spermatocytes. Differential expression analysis revealed an outburst of TE expression in the 5DCR spermatocytes. In 3DCR, only 8.5% of the TE transcripts were dysregulated (Fig. 6E); however, in 5DCR, this percentage increased to 68.4%, of which up to 66.6% were constituted by upregulated transcripts alone (Fig. 6F). The major disturbances were seen in the expression of the LTRs (50.4%), followed by DNA transposons (21.9%), LINE (17.7%), SINE (4.8%), and unknown elements (3.8%). In-depth analysis of the repeat families identified that the ERVK and L1 families were majorly upregulated in heat-stressed spermatocytes (Fig. 6G). We also carried out similar analysis in round spermatids as well, where only 1.25% TE transcripts in 3DCR and 4.98% in 5DCR were dysregulated (data not presented).

### Upregulation of piRNA machinery and heat shock proteins with heat stress

**Fig. 7:**
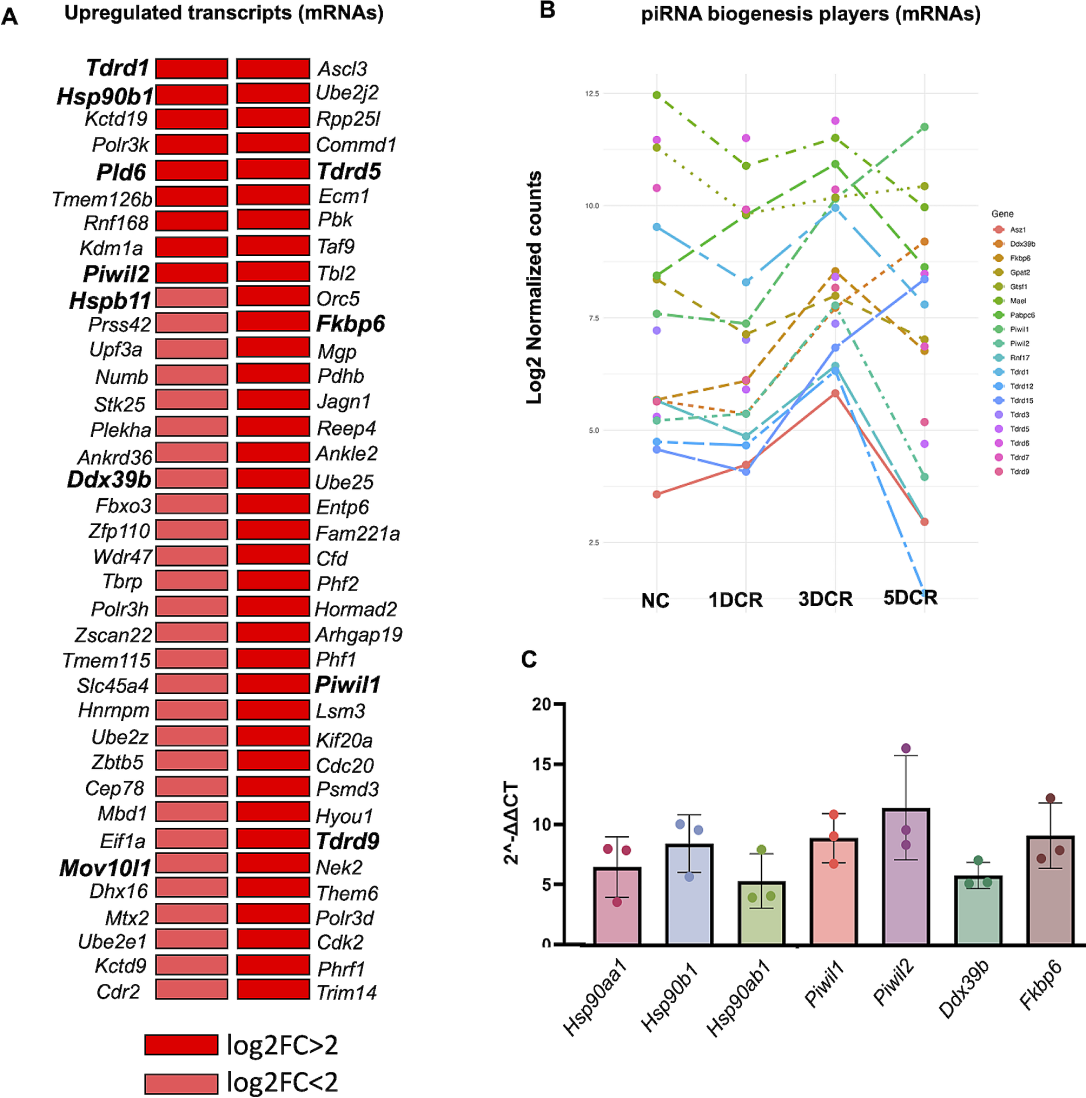
Differential gene expression analysis of mRNAs in heat stress. The list of upregulated transcripts in 3DCR spermatocytes (**A**), upregulation of piRNA biogenesis players  (**B**), RT-PCR validation of the NGS data (**C**)

The analysis of transcriptome data revealed that the piRNA machinery got upregulated under heat stress. The comparison of normal spermatocytes with 1DCR spermatocytes showed upregulations of heat shock transcripts, i.e., *Hspa5* ,*Hsph1*, *Hsp90ab1*, *Hspd1* as an immediate response to the stress stimuli (Fig. 7A); however, other piRNA biogenesis players were not altered. Interestingly, in the 3DCR spermatocytes, along with heat shock protein upregulation, there was upregulation of a number of piRNA biogenesis players, such as *Piwil1*, *Piwil2*, *Tdrd1*, *Tdrd5, Ddx39B*, *Fkbp6*, and *Mov10l1* (Fig. 7B). In 5DCR, the heat shock proteins and PIWIL1 were upregulated, but other players were downregulated, as if the damage control machinery had failed to sustain stress. We also performed RT-PCR expression comparisons in NC and 3DCR spermatocytes for the consistently upregulated transcripts for validation purposes and gained confidence in our differential expression findings (Fig. 7C). In round spermatids, we observed upregulation of heat shock proteins but the piRNAs biogenesis players were not dysregulated.

### Target prediction for piRNAs

piRNAs are known to target not onlty TE elements, but also mRNAs. Therefore, we predicted targets for differentially expressed piRNAs. We used a minimum of 16 nucleotide complementarity rule for piRNA target prediction. Target prediction analysis was undertaken for spermatocyte-specific clusters to understand the general functions in spermatocytes and for differentially expressed piRNAs to understand the effect of heat stress mediated by piRNAs.

**Fig. 8:**
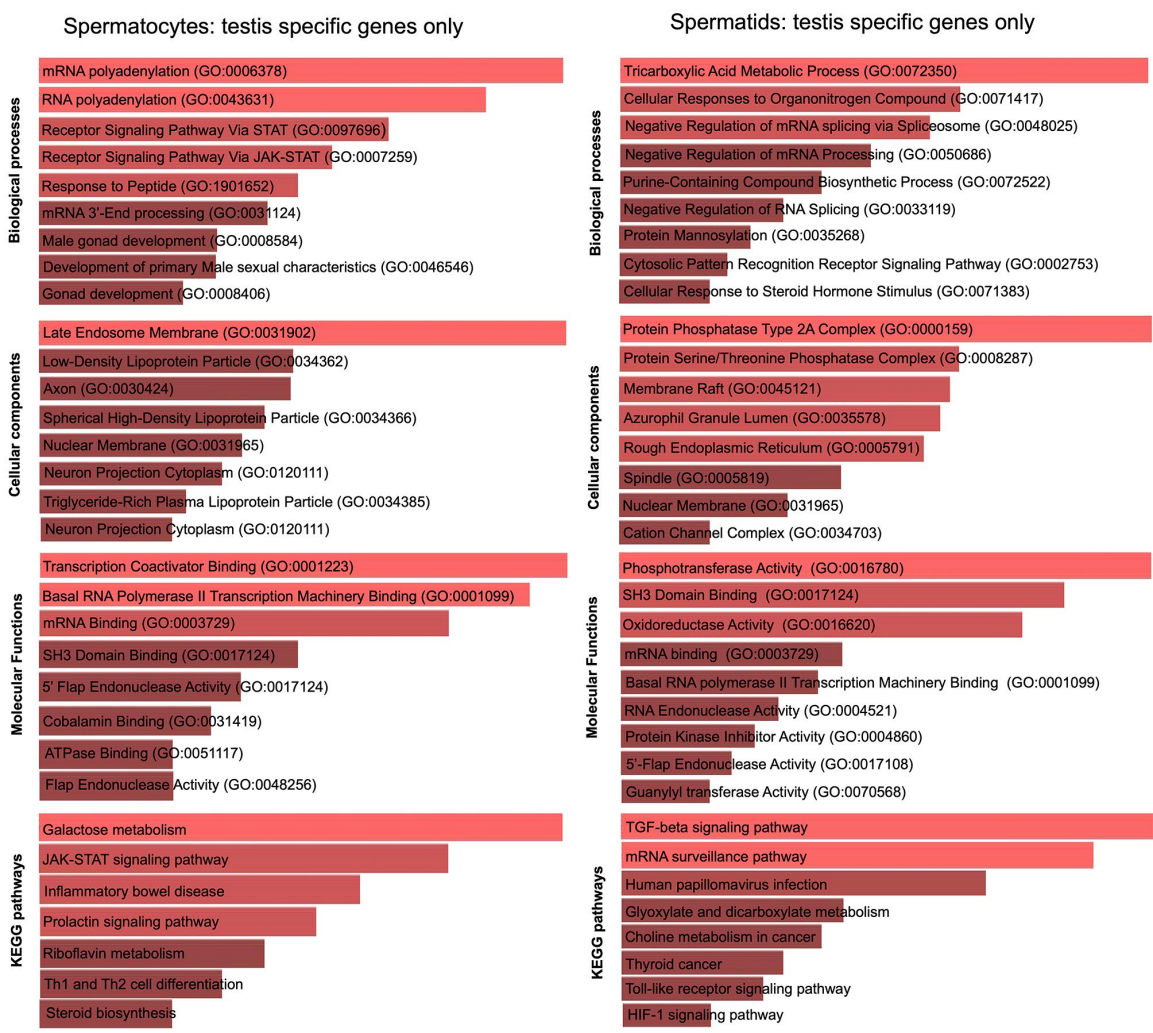
piRNA targets downstream annotation: Gene ontology (GO) and Kyoto Encyclopedia of Genes and Genomes (KEGG) pathway enrichment analyses for piRNA targets

First, we identified spermatocyte-specific clusters with high stringency. Miranda predicted 257 target transcripts on the basis of the 3’UTR complementarity and 310 target transcripts on the basis of the CDS complementarity. There were negligible piRNAs that targeted the 5’UTR of transcripts. The numbers of 5’UTR, CDS, and 3’UTR targets corresponding to each spermatocyte specific piRNA cluster are presented in Supplementary Fig. 3. For the predicted targets, we carried out gene ontology (GO) and Kyoto Encyclopaedia of Genes and Genomes (KEGG) pathway enrichment analyses separately for 3’UTR and CDS targets, which identified genes related to JAK-STAT signalling, TGF-beta signalling, virus infection, mRNA surveillance pathways, etc. Gene ontology analysis covered biological processes that involved mRNA polyadenylation, mRNA 3’end processing, negative regulation of mRNA splicing, gonad development, JAK-STAT signalling, etc. The molecular functions of the predicted targets involved mRNA binding, RNA endonuclease activity, phosphotransferase activity, RNA pol II binding, etc. (Fig. 8).

**Fig. 9:**
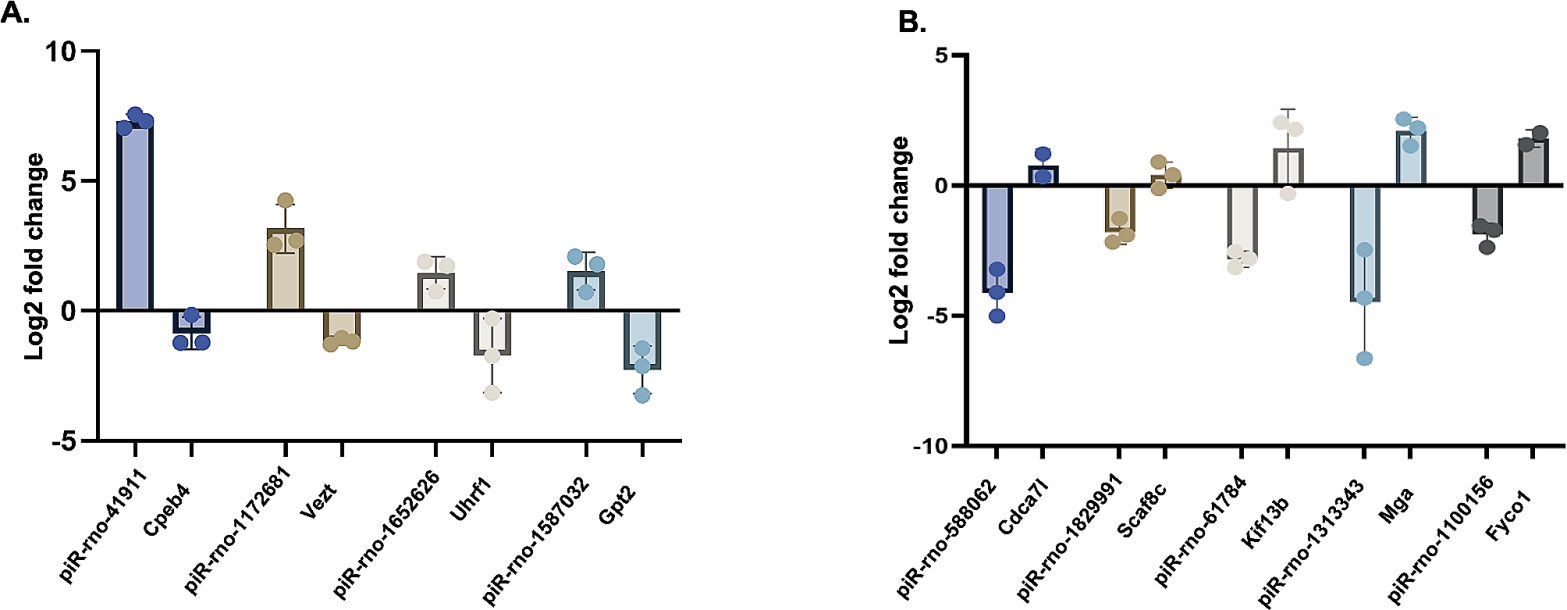
Representative piRNA-mRNA pairs showing inverse relation in transcriptome analysis. Upregulated piRNAs and their downregulated target expression in heat stressed spermatocytes (**A**). Downregulated piRNAs and their upregulated targets expression in heat stressed spermatocytes (**B**)

Next, we focussed on the targets of the upregulated and downregulated piRNAs shared by the 3DCR and 5DCR pachytene spermatocytes. The pairs (piRNAs and targets) with inverse relations were further looked at for their roles in spermatogenesis and transposon regulation. The piRNA/PIWI (piRISC) complex interacts with CAF1 deadenylase to stimulate the deadenylation of transcripts [26]. Therefore, we also looked for alterations in the predicted targets in the MIWI and CAF1 knockdown mouse testes datasets. We shortlisted the targets on the basis of their inverse relation with piRNAs and their upregulation in MIWI and CAF1 knockdown datasets. The piRNAs, piR-rno-41911, piR-rno-1172681, piR-rno-1652626, piR-rno-1587032 were upregulated and their predicted targets, i.e., Cpeb4, Vezt, Uhrf1, and Gpt2, respectively, were downregulated in heat-stressed spermatocytes (Fig. 9). Similarly, from the downregulated piRNAs, we shortlisted piRNA-mRNA pairs showing inverse relationship, which included piR-rno-1829991, piR-rno-1929310, piR-rno-61784, piR-rno-1313343, and piR-rno-1100156 and their upregulated targets Scaf8, Napa, Kif13b, Mga, and Fyco1, respectively (Fig. 9).

### In vitro validation of piRNA targets

To validate piRNA targets in an in vitro system, we first tested the available germ cell lines, such as GC1 (spermatogonia) and GC2 (spermatocytes) for the presence of piRNA machinery. We found low levels of MILI and MIWI2 RNA, but MIWI was not present in both these cell lines. In addition to the MIWI transcripts, we also checked for the presence of GTSF1, which is considered an essential factor for secondary piRNA biogenesis, and Mael, which is located in nuage and is required for transposon silencing. We found the presence of GTSF1 in both cell lines; however, Mael was not found (Supplementary Fig. 4).

Previously, many studies have carried out synthetic piRNA experiments in the GC2 cell line; therefore, we used this cell line for in vitro experiments. We picked a top upregulated (piR-rno-41911) and a top downregulated piRNA (piR-rno-588062) for in vitro validation of their relationship with their targets. As detailed in the methods section, we carried out this experiment in two ways; with and without MIWI transfection.

In the first round of experiments, we did not use MIWI along with piRNAs. Briefly, GC2 cells (CRL-2196™, ATCC) at 70% confluency were transfected with synthetic piRNAs. The transfected cells observed under a microscope after a DPBS wash displayed cy3 positivity, which confirmed piRNA transfection (data not shown). We also checked the levels of specific piRNAs in transfected and non-transfected cells to confirm the stability of the transfected sequences using specific stem loop primers (data not shown). In both transfection experiments, the targets showed statistically insignificant variations in expressions. In the second round of experiments, MIWI expressing GC2 cells were transfected with piRNAs and control sequences. However, all the targets showed statistically insignificant variations in their expression. No effect on target expression in in vitro experiments could be due to the use of single piRNAs in these experiments against the clustering nature of their biological functions or the absence of several other components of the PIWI complex that are required for target engagement in GC2 cells.

## Discussion

For piRNA prediction, we considered the reads with lengths of 24–35 nt and 5’U bias, which is a hallmark characteristic of piRNAs. We observed high numbers of piRNAs in spermatocytes (28,800) and spermatids (19951). The reads corresponding to 29 and 30 nucleotides constituted the major fraction in both spermatocytes and spermatids. However, another major peak seen at 26 nucleotides in the case of spermatocytes disappeared in spermatids. A previous study on piRNA sequence length showed that the major peak in spermatocytes was at 26 nucleotides, which changed to 30 in the case of spermatids [25]. In both cell types, about 80% of piRNAs were non-repeat derived. A previous study on piRNAs in testicular germ cells showed that piRNAs in spermatocytes and spermatids switched from repeat-derived and mRNA-derived piRNAs in spermatogonia to intergenic-derived piRNAs [25]. Further mapping of the non-repeat derived sequences revealed that the majority of them originated from intergenic regions. We observed ping-pong amplification in spermatocytes, but not in spermatids. Ping-pong signatures have been noted to be more prominent in spermatogonia in comparison to spermatocytes. This may be because of the absence of Ago3 in spermatocytes [27]. We believe that ping-pong amplification may diminish further due to the absent/low levels of Ago3 in spermatids. piRNA production, particularly that of intergenic ones, may not be ping-pong dependent.

Next, we asked how piRNAs changed under heat stress. In addition to significant alterations in piRNA levels under heat stress, about 18,000 and 10,000 new piRNAs appeared in spermatocytes and spermatids, respectively. As is true for normal spermatocytes, a major chunk (61.50%) of these new piRNAs was intergenic-derived. Heat stressed spermatocytes also showed increased ping-pong activity, which was not seen in spermatids. In addition to piRNAs, TE transcripts showed significant upregulation in 3DCR spermatocytes and 5DCR showed TE outburst, which suggests that piRNA upregulation may be a rescue mechanism as a result of increased TE activity. Previous studies have shown that piRNAs are dynamic as they switch from mRNA-derived and repeat-derived piRNAs in the 10-dpp testis to intergenic-derived piRNA in the adult testis [25]. We observed a similar dynamism in heat stress in the form of piRNA upregulation and the generation of ∼ 38.48% new piRNAs in heat stressed spermatocytes only. These may be reserved piRNAs that are expressed under stress conditions only. Looking at this, piRNAs are also worth studying under other testicular stress conditions. A similar previous study on the mouse testicular heat stress model showed significant upregulation of piRNAs and associated transcripts [28], though this study conducted analysis on whole testicular tissue. We have conducted the first investigation on heat-induced piRNA alterations in testicular germ cells.

In addition to piRNA alterations, many piRNA biogenesis players were significantly upregulated in the 3DCR pachytene spermatocytes, some of which maintained upregulation even in the 5DCR stage. HSP90 is the most abundant chaperone, which stabilizes the conformational state of proteins against heat stress to maintain homeostasis. Studies on Drosophila and other models have implicated HSP90, Hop, FKBP6, HSP70 and other chaperones in piRNA biogenesis and TE activity [15, 29–32]. In addition to this, studies on Drosophila and mammals have shown that HSP90 is important for spermatogenesis, piRNA production, and repression of retrotransposons in germ cells [31, 32]. From the above, it can be concluded that the piRNA machinery goes hand-in-hand with heat shock proteins in normal as well as stress exposed germ cells, probably to protect genome integrity by repressing transposable elements. It is probably because of this activation of piRNAs that TE activity was restricted in the case of the 3DCR stage (8.5% of TE transcripts), which showed an outburst upon continued stress in the 5DCR stage (66.6% of the TE transcripts, log2FC>1.5). This may indicate the failure of the stress management machinery upon continued exposure to stress. A similar study on stress exposure in Drosophila reported a significant increase in transcripts of all TEs in the ovaries and testes [15].

Next, we asked how these changes in piRNA expressions are related to transcriptome changes in heat stress. Upon target prediction, piRNAs and mRNA transcripts with inverse relations in the two data sets were explored for their role in spermatogenesis. Among some of the targets of piRNAs in heat stress, CPEB4 is an RNA-binding protein that mediates polyadenylation and translation of meiotic mRNAs, SCD2 (Stearoyl Coenzyme A desaturase 2) catalyses the conversion of polyunsaturated fatty acids (PUFAs) to monounsaturated fatty acids (MUFAs) in the testis [33], which facilitates membrane fluidity for sperm motility, flexibility, receptor functions, acrosome reaction, and fertilization [34]. Among other target pairs, piR-rno-1172681 targeted Vezt (Vezatin), which is an important protein of cell-cell adherens junctions [35], piR-rno-1652626 targeted Uhrf1 (Ubiquitin Like With PHD and Ring Finger Domains 1), which is involved in retrotransposon silencing and epigenetic pathways [36], piR-rno-1587032 targeted GPT2 (Glutamic Pyruvic Transaminase 2), which promotes tumorigenesis [37]. Among the downregulated piRNAs, one of the top downregulated piRNAs, i.e., piR-rno-588062 targets Cdca7l (cell division cycle associated 7 like protein), which is involved in positive regulation of cell proliferation [38]. Other downregulated piRNAs included piR-rno-61784, which targets Kif13b, a protein involved in cell polarity and adhesion [39], piR-rno-1829991 targets Scaf8, which prevents early mRNA termination during transcription [40]. All of these targets were upregulated in MIWI and CAF1 knockdown animals [26], establishing their regulation by piRNAs. Enrichment analysis for the predicted mRNA targets revealed enrichment of the JAK STAT cascade, Herpes simplex virus infection, human papilloma virus infection, etc. Interestingly, the JAK STAT pathway is instrumental for cell survival in response to stress, and the virus interaction pathways suggest the participation of piRNAs in the cellular stress response.

In vitro functional assays using one of the top most upregulated (piR-rno-41911) and one of the top downregulated (piR-rno-588062) did not show statistically significant alterations in their targets. There could be several reasons behind this observation. The foremost is that GC2 cells do not express the full machinery required for piRNA functions. In these cells, we found GTSF1 to be present, MILI and MIWI2 to be present at low levels, but MIWI and Mae1 were absent. We tried to supplement this machinery using a MIWI expression clone, but still the target gene expression did not change significantly. Another major reason behind this could be the clustering nature of piRNA functions. Most piRNAs function as clusters, and the presence/absence of a single piRNA is less likely to bring significant alteration in its target gene. Further, not everything is known about the specific targeting mechanisms of piRNAs. Even in the case of in vivo experiments targeting full piRNA clusters, results have varied highly significantly. For example, a recent knockout study for the mouse pi6 cluster (Chr6: 127776075–127841890), which corresponds to the rat bidirectional cluster on chromosome 4 (Chr4:160350699–160416076), disrupted male fertility, characterized by defective capacitation and failed penetration of the zona pellucida by mutant sperm [41]. Similarly, another cluster knockout of pi18 (Chr18: 67029446:67084379) resulted in sperm head dysmorphology, acrosome overgrowth, and sterility in male mice [42]. However, the knockout of the biggest piRNA cluster, i.e., pi17 (Chr17:27288275–27367483), which corresponds to the bidirectional cluster on Chr 20 (Chr20:6635795–6674926) of rats, had no effect on male fertility [41].

In conclusion, this study profiled the changes in piRNAs, transposable elements, and mRNAs in pachytene spermatocytes in response to testicular heat stress. We conclude that heat shock proteins go hand-in-hand with the piRNA biogenesis machinery elements, such as PIWIL1, TDRD5, PIWIL2, etc. to regulate piRNA production under heat stress. However, piRNA biogenesis players were not upregulated in round spermatids. Heat stress appeared to increase the TE activity moderately in the 3DCR stage (8.5% of TE transcripts) and profusely in the 5DCR stage (66.6% of the TE transcripts). With regard to their origin, ∼ 80% of the pachytene piRNAs were non-repeat derived and only 20% were repeat-derived. Among non-repeat derived piRNAs, most of them belonged to intergenic regions. A cumulative analysis of all pachytene piRNAs predicted 63 piRNA clusters. The JAK-STAT pathway surfaced as a major target of these clusters, while other targets included Herpes simplex virus infection and human papilloma virus infection-related genes, all of which suggested the activation of the stress response. The major biological processes regulated by the pachytene piRNAs were RNA processing, polyadenylation, and splicing. In a nutshell, piRNAs are critical to the pachytene spermatocytes and their upregulation in heat stress is probably a stress management mechanism to lower DNA damage, maintain genome integrity, and regulate transposable elements to maintain testicular homeostasis during stress. This is the first study on rat testicular germ cells under heat stress. In the initial phase of stress, piRNAs, piRNA machinery, and heat shock proteins are activated to deal with low levels of stress, which is followed by a rescue approach in prolonged stress period, followed by high TE activity to allow genetic mutations for survival and adaptability. These responses are perhaps managed in a manner to cope with low levels of stress and avoid undue triggering of the stress response, timely downregulation of the response, and genetic adaptability.

### Electronic supplementary material

Below is the link to the electronic supplementary material.

**Supplementary Material 1: Supplementary Fig. 1:** The distribution of the read count percentage corresponding to the read length of 24-35 nucleotides in pachytene spermatocytes and round spermatids

**Supplementary Material 2: Supplementary Fig. 2:** The set of piRNAs derived from lncRNAs. The height of the bar corresponds to their abundances

**Supplementary Material 3: Supplementary Fig. 3:** mRNAs targets of the piRNA clusters. The length of the bar corresponds to the number of genes showing complementarity (minimum 16 nt to maximum full length complementarity) to the target

**Supplementary Material 4: Supplementary Fig. 4:** Expression of Piwi transcripts in testis, GC1 and GC2 cells

**Supplementary Material 5: Supplementary Table 1:** Oligonucleotide sequences used in the study. **Supplementary Table 2:** proTRAC predicted piRNA clusters in pachytene spermatocytes and round spermatids. The common clusters between the two cell types are highlighted in red. **Supplementary Table 3:** proTRAC predicted unique piRNA clusters in heat stressed pachytene spermatocytes

## Data Availability

No datasets were generated or analysed during the current study.
