# Supplementary figures and images for: Heat stress induced piRNA alterations in pachytene spermatocytes and round spermatids
Source: Reprod Biol Endocrinol. 2024 Jul 24;22:87. doi: 10.1186/s12958-024-01249-z (PMC11267754; doi:10.1186/s12958-024-01249-z)

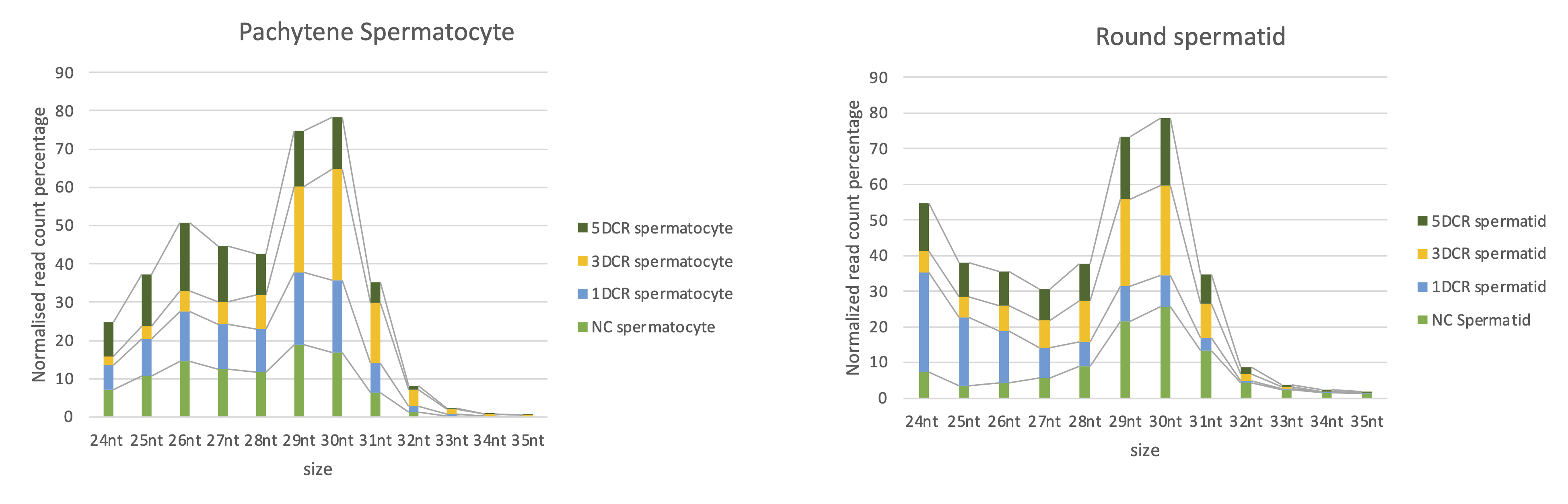

Supplement: Supplementary file 1 — Supplementary Material 1: Supplementary Fig. 1: The distribution of the read count percentage corresponding to the read length of 24-35 nucleotides in pachytene spermatocytes and round spermatids [file 12958_2024_1249_MOESM1_ESM.png]

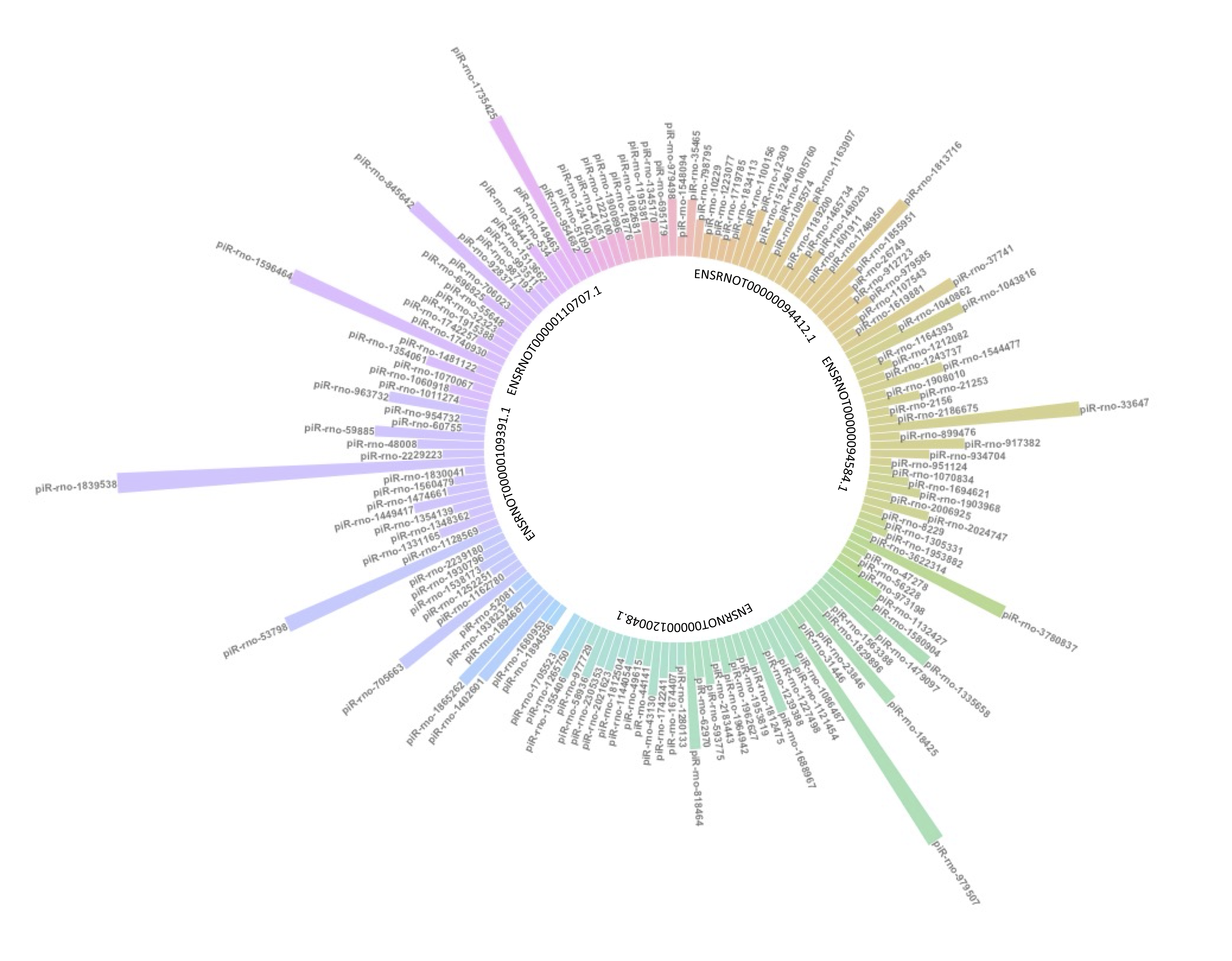

Supplement: Supplementary file 2 — Supplementary Material 2: Supplementary Fig. 2: The set of piRNAs derived from lncRNAs. The height of the bar corresponds to their abundances [file 12958_2024_1249_MOESM2_ESM.png]

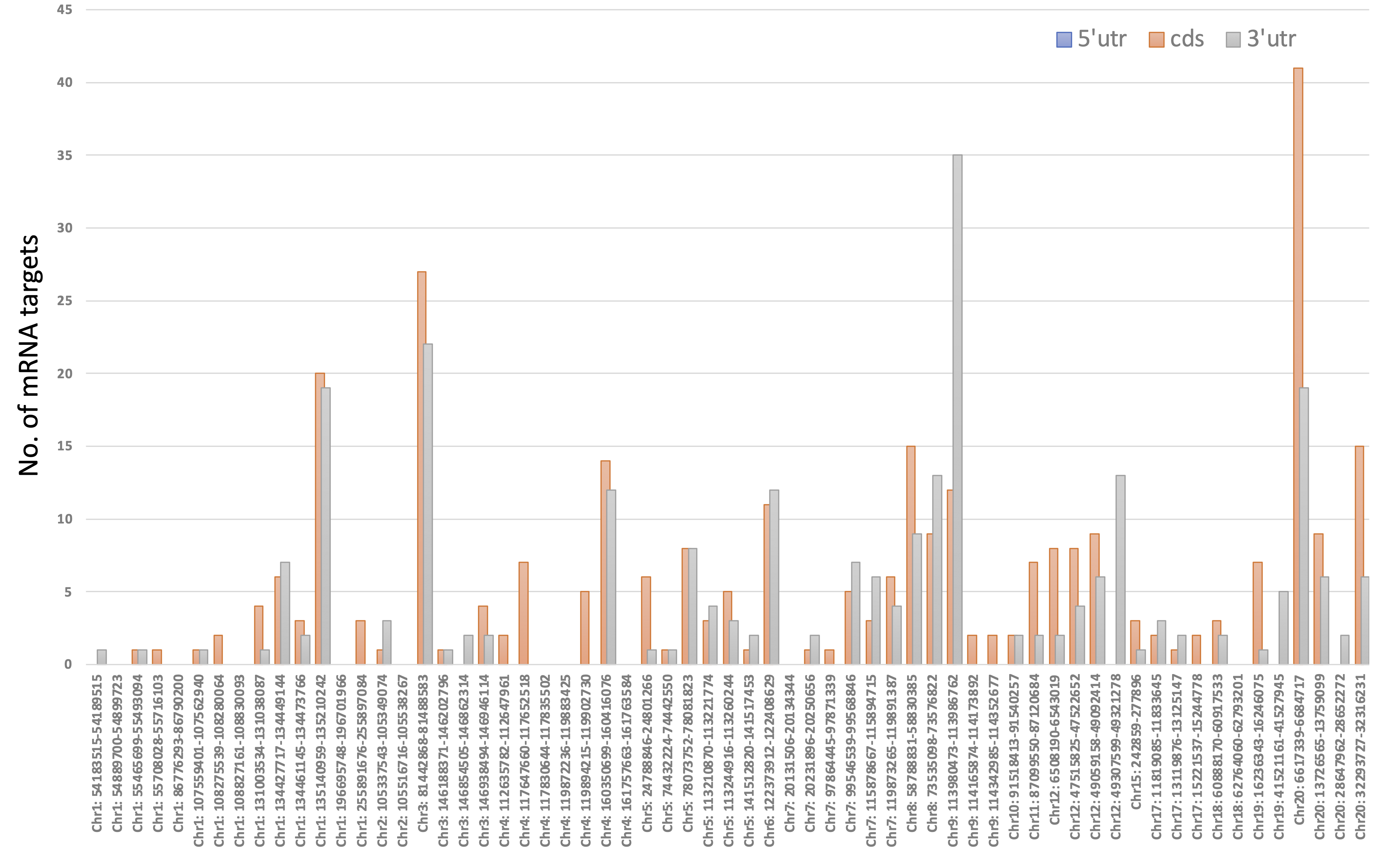

Supplement: Supplementary file 3 — Supplementary Material 3: Supplementary Fig. 3: mRNAs targets of the piRNA clusters. The length of the bar corresponds to the number of genes showing complementarity (minimum 16 nt to maximum full length complementarity) to the target [file 12958_2024_1249_MOESM3_ESM.png]

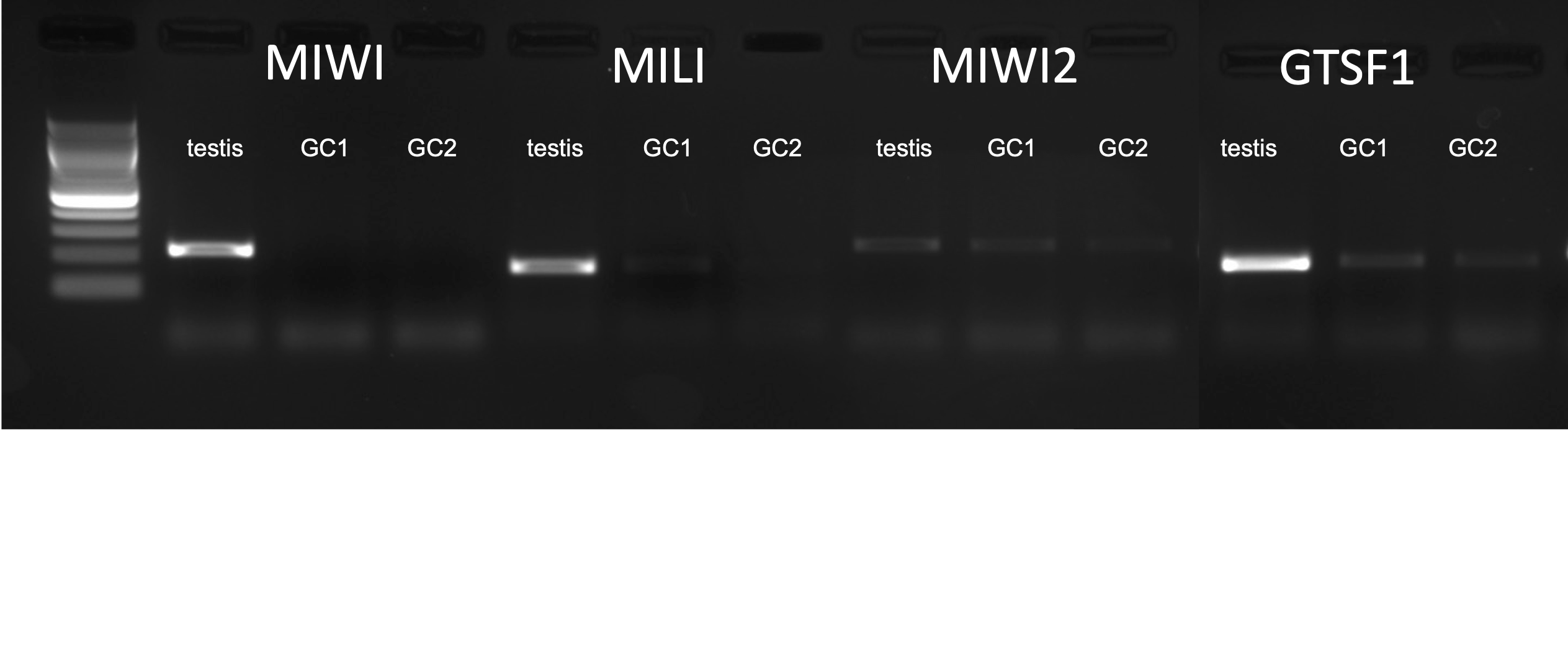

Supplement: Supplementary file 4 — Supplementary Material 4: Supplementary Fig. 4: Expression of Piwi transcripts in testis, GC1 and GC2 cells [file 12958_2024_1249_MOESM4_ESM.png]
