# Supplementary material for: Heat stress induced piRNA alterations in pachytene spermatocytes and round spermatids
Source: Reprod Biol Endocrinol. 2024 Jul 24;22:87. doi: 10.1186/s12958-024-01249-z (PMC11267754; doi:10.1186/s12958-024-01249-z)
Supplement: Supplementary file 5 — Supplementary Material 5: Supplementary Table 1: Oligonucleotide sequences used in the study. Supplementary Table 2: proTRAC predicted piRNA clusters in pachytene spermatocytes and round spermatids. The common clusters between the two cell types are highlighted in red. Supplementary Table 3: proTRAC predicted unique piRNA clusters in heat stressed pachytene spermatocytes [file 12958_2024_1249_MOESM5_ESM.docx]

**Supplementary Table 1: Oligonucleotide sequences used in the study.**

| **Gene Name/piRNA ID** | **RT primer** | **Forward primer** | **Reverse primer** |
| --- | --- | --- | --- |
| ***piR-rno-41911*** | 5’/Cy3/GACTCTTAGCGGTGGATCACTCGGCTCGTGmC3’ | | |
| **piR-rno-588062** | 5'/Cy3/CAGCTCTGGCTTTGTGCTGAACACACmC3 | | |
| *piR-rno-41911* | GTCGTATCCAGTGCAGGGTCCGAGGTATTCGCACTGGATACGACGCACGA | AACAAGGACTCTTAGCGGTGG | GTCGTATCCAGTGCAGGGT |
| *piR-rno-588062* | GTCGTATCCAGTGCAGGGTCCGAGGTATTCGCACTGGATACGACGGTGTG | AACAAGCAGCTCTGGCTTTG | GTCGTATCCAGTGCAGGGT |
| *Scd2* | Random hexamers | AAGCAGCTCTCTTCTTAGCCTGC | CACGAGGCAAGGACTGTTAGAAAACT |
| *Cpeb4* | Random hexamers | CTCACACTTTGGAAGATCTTTGCAGTG | GCAAATGACCAAAACCTCTTTTATTCC |
| *Cdca7l* | Random hexamers | GTGCTTCCGTTCCAAATACTTCA | ACTGCTTCTTCTTGTGGACCTG |
| *Hsp90ab1* | Random hexamers | ATGTCCCTCATCATCAACACTTTCTACT | AGTTATTTATGAGGTCAGCCTTGGTCA |
| *Hsp90b1* | Random hexamers | TTGTGAGAGCTGACGATGAAGTC | GTTAACTTCAGCTTGGAAGGCG |
| *Hsp90aa1* | Random hexamers | TTGGTTGCTGAGAAAGTGACTGTCAT | GAGAGTAATGGGGTAGCCAATAAACTGAG |
| *Ddx39b* | Random hexamers | AACAGCAGTGTTTGTCCTGGC | TTATTTCGGGCCAGGGCTAGAATT |
| *Fkbp6* | Random hexamers | GACCGAGGTGTGCTGAAAGATAT | AGCATAGGTTGGCTTGAACAGG |
| *Miwi* | Random hexamers | TGGACGGAATTATTACAACCCAAGTG | TATGAGCTCCTTGGACACTTGCTC |
| *Mili* | Random hexamers | TTAGGCAGAGGAGTTCTAGGTCGA | CTCAGAACTACCTCTTCCCAGCATT |

**Supplementary Table 2: proTRAC predicted piRNA clusters in pachytene spermatocytes and round spermatids. The common clusters between the two cell types are highlighted in red.**

| No. | **Pachytene spermatocytes piRNA clusters** | | | **Round spermatids piRNA clusters** | | |
| --- | --- | --- | --- | --- | --- | --- |
|  | **Chromosome: coordinates** | **strand** | **directionality** | **Chromosome: coordinates** | **strand** | **directionality** |
| 1 | Chr1: 54183515-54189515 | minus | unidirectional | Chr1: 14133597-14153865 | minus | unidirectional |
| 2 | Chr1: 54889700-54899723 | minus | unidirectional | Chr1: 54199608-54214065 | minus | unidirectional |
| 3 | Chr1: 55465699-55493094 | plus | unidirectional | Chr1: 55466470-55493094 | plus | unidirectional |
| 4 | Chr1: 55708028-55716103 | plus | unidirectional | Chr1: 108275539-108280064 | plus | unidirectional |
| 5 | Chr1: 86776293-86790200 | plus:minus | bidirectional | Chr1: 131004528-131037250 | minus:plus | bidirectional |
| 6 | Chr1:107559401-107562940 | minus | unidirectional | Chr1: 134427717-134449188 | minus:plus | bidirectional |
| 7 | Chr1:108275539-108280064 | plus | unidirectional | Chr1: 134461145-134473670 | plus | unidirectional |
| 8 | Chr1:108827161-108830093 | minus | unidirectional | Chr1: 135142987-135210242 | minus:plus | bidirectional |
| 9 | Chr1:131003534-131038087 | minus:plus | bidirectional | Chr1: 255891676-255896846 | minus | unidirectional |
| 10 | Chr1:134427717-134449144 | minus:plus | bidirectional | Chr10: 91521997-91540257 | plus | unidirectional |
| 11 | Chr1:134461145-134473766 | plus | unidirectional | Chr12: 6508190-6542777 | minus | unidirectional |
| 12 | Chr1:135140959-135210242 | minus:plus | bidirectional | Chr12: 47515865-47522478 | plus | unidirectional |
| 13 | Chr1:196695748-196701966 | minus | unidirectional | Chr12: 49060964-49092414 | minus:plus | bidirectional |
| 14 | Chr1:255891676-255897084 | minus | unidirectional | Chr12: 49307599-49321842 | plus | unidirectional |
| 15 | Chr10: 91518413-91540257 | plus | unidirectional | Chr15: 252345-278727 | minus | unidirectional |
| 16 | Chr11: 87099550-87120684 | minus:plus | bidirectional | Chr17: 11790417-11800629 | minus | unidirectional |
| 17 | Chr12: 6508190-6543019 | minus | unidirectional | Chr17: 11829795-11834293 | minus | unidirectional |
| 18 | Chr12: 47515825-47522652 | plus | unidirectional | Chr17: 14432913-14435281 | minus | unidirectional |
| 19 | Chr12: 49059158-49092414 | minus:plus | bidirectional | Chr17: 15221537-15244778 | plus | unidirectional |
| 20 | Chr12: 49307599-49321278 | plus | unidirectional | Chr18: 60888170-60917625 | minus | unidirectional |
| 21 | Chr15: 242859-277896 | minus | unidirectional | Chr18: 62764060-62793293 | minus | unidirectional |
| 22 | Chr17: 11819085-11833645 | minus | unidirectional | Chr19: 41521564-41527945 | minus | unidirectional |
| 23 | Chr17: 13119876-13125147 | minus | unidirectional | Chr2: 105337947-105349074 | minus | unidirectional |
| 24 | Chr17: 15221537-15244778 | plus | unidirectional | Chr2: 105370121-105380740 | minus | unidirectional |
| 25 | Chr18: 60888170-60917533 | minus | unidirectional | Chr2: 105517120-105538267 | minus | unidirectional |
| 26 | Chr18: 62764060-62793201 | minus | unidirectional | Chr2: 105559618-105570237 | minus | unidirectional |
| 27 | Chr19: 16236343-16246075 | plus | unidirectional | Chr20: 6618730-6684719 | minus:plus | bidirectional |
| 28 | Chr19: 41521161-41527945 | minus | unidirectional | Chr20: 13727741-13759099 | minus | unidirectional |
| 29 | Chr2:105337543-105349074 | minus | unidirectional | Chr20: 32288883-32316231 | plus | unidirectional |
| 30 | Chr2:105516716-105538267 | minus | unidirectional | Chr3: 81442868-81488583 | plus | unidirectional |
| 31 | Chr20: 6617339-6684717 | minus:plus | bidirectional | Chr3: 145487678-145492468 | plus | unidirectional |
| 32 | Chr20: 13726565-13759099 | minus | unidirectional | Chr3: 145602814-145612502 | plus | unidirectional |
| 33 | Chr20: 28647962-28652272 | plus | unidirectional | Chr3: 146189705-146202796 | minus | unidirectional |
| 34 | Chr20: 32293727-32316231 | plus | unidirectional | Chr3: 146940002-146946114 | plus | unidirectional |
| 35 | Chr3: 81442868-81488583 | plus | unidirectional | Chr4: 77401272-77411969 | minus | unidirectional |
| 36 | Chr3:146188371-146202796 | minus | unidirectional | Chr4: 84979928-84989462 | plus | unidirectional |
| 37 | Chr3:146854505-146862314 | minus | unidirectional | Chr4: 116827852-116830841 | minus | unidirectional |
| 38 | Chr3:146938494-146946114 | plus | unidirectional | Chr4: 116883440-116886476 | minus | unidirectional |
| 39 | Chr4:112635782-112647961 | minus | unidirectional | Chr4: 119896120-119902506 | minus | unidirectional |
| 40 | Chr4:117647660-117652518 | minus | unidirectional | Chr4: 160351448-160407435 | minus:plus | bidirectional |
| 41 | Chr4:117830644-117835502 | minus | unidirectional | Chr4: 161757663-161763004 | plus | unidirectional |
| 42 | Chr4:119872236-119883425 | minus | unidirectional | Chr5: 51037110-51040445 | plus:minus | bidirectional |
| 43 | Chr4:119894215-119902730 | minus | unidirectional | Chr5: 74433008-74440610 | minus | unidirectional |
| 44 | Chr4:160350699-160416076 | minus:plus | bidirectional | Chr5: 78073769-78081518 | plus | unidirectional |
| 45 | Chr4:161757663-161763584 | plus | unidirectional | Chr5: 113210870-113219151 | minus | unidirectional |
| 46 | Chr5: 24788846-24801266 | minus | unidirectional | Chr5: 113244896-113260244 | minus | unidirectional |
| 47 | Chr5: 74432224-74442550 | minus | unidirectional | Chr5: 141514154-141517455 | minus | unidirectional |
| 48 | Chr5: 78073752-78081823 | plus | unidirectional | Chr6: 122373912-122409400 | minus-plus | bidirectional |
| 49 | Chr5:113210870-113221774 | minus | unidirectional | Chr7: 20131506-20134344 | minus | unidirectional |
| 50 | Chr5:113244916-113260244 | minus | unidirectional | Chr7: 20232579-20250656 | plus | unidirectional |
| 51 | Chr5:141512820-141517453 | minus | unidirectional | Chr7: 99540601-99569204 | minus:plus | bidirectional |
| 52 | Chr6:122373912-122408629 | minus:plus | bidirectional | Chr7: 115874558-115893242 | plus | unidirectional |
| 53 | Chr7: 20131506-20134344 | minus | unidirectional | Chr7: 119873265-119888695 | minus | unidirectional |
| 54 | Chr7: 20231896-20250656 | plus | unidirectional | Chr8: 58782311-58830385 | minus:plus | bidirectional |
| 55 | Chr7: 97864445-97871339 | minus | unidirectional | Chr8: 73535098-73588370 | minus:plus | bidirectional |
| 56 | Chr7: 99546539-99568846 | minus:plus | bidirectional | Chr9: 114002834-114009960 | plus | unidirectional |
| 57 | Chr7:115878667-115894715 | plus | unidirectional | ChrX: 100582280-100588088 | plus | unidirectional |
| 58 | Chr7:119873265-119891387 | minus | unidirectional |  |  |  |
| 59 | Chr8: 58788831-58830385 | minus | unidirectional |  |  |  |
| 60 | Chr8: 73535098-73576822 | minus:plus | bidirectional |  |  |  |
| 61 | Chr9:113980473-113986762 | minus:plus | bidirectional |  |  |  |
| 62 | Chr9:114165874-114173892 | minus:plus | bidirectional |  |  |  |
| 63 | Chr9:114342985-114352677 | plus | unidirectional |  |  |  |

**Supplementary Table 3: proTRAC predicted unique piRNA clusters in heat stressed pachytene spermatocytes.**

| No. | **Heat stress unique piRNA clusters in pachytene spermatocytes** | | |
| --- | --- | --- | --- |
|  | **Chromosome: coordinates** | **strand** | **directionality** |
| 1 | Chr1: 54479315-54492232 | plus | unidirectional |
| 2 | Chr1: 55399142-55406426 | plus | unidirectional |
| 3 | Chr1: 55776659-55789914 | plus | unidirectional |
| 4 | Chr1: 97776790-97781151 | minus | unidirectional |
| 5 | Chr1: 117083130-117091157 | minus | unidirectional |
| 6 | Chr1: 194801253-194804518 | minus | unidirectional |
| 7 | Chr17: 11757124-11762217 | minus | unidirectional |
| 8 | Chr17: 11789262-11801318 | minus | unidirectional |
| 9 | Chr17: 11829795-11839008 | minus | unidirectional |
| 10 | Chr17: 15004145-15007275 | plus | unidirectional |
| 11 | Chr2: 105859015-105868327 | minus | unidirectional |
| 12 | Chr2: 128884854-128901713 | minus | unidirectional |
| 13 | Chr3: 145599554-145612502 | plus | unidirectional |
| 14 | Chr4: 116883436-116886476 | minus | unidirectional |
| 15 | Chr5: 153976616-153979305 | plus | unidirectional |
| 16 | Chr7: 70993556-70998443 | plus | unidirectional |
| 17 | Chr8: 48482866-48486036 | minus | unidirectional |
| 18 | Chr9: 100148000-100152496 | minus | unidirectional |
| 19 | Chr9: 113964183-113970166 | plus | unidirectional |
| 20 | Chr9: 114003990-114010782 | plus | unidirectional |
